# Cadmium, Lead, and Mercury in Relation to Reproductive Hormones and Anovulation in Premenopausal Women

**DOI:** 10.1289/ehp.1003284

**Published:** 2011-05-04

**Authors:** Anna Z. Pollack, Enrique F. Schisterman, Lynn R. Goldman, Sunni L. Mumford, Paul S. Albert, Robert L. Jones, Jean Wactawski-Wende

**Affiliations:** 1 *Eunice Kennedy Shriver* National Institute of Child Health and Human Development, Epidemiology Branch, Rockville, Maryland, USA; 2 Johns Hopkins Bloomberg School of Public Health, Department of Epidemiology, Baltimore, Maryland, USA; 3 *Eunice Kennedy Shriver* National Institute of Child Health and Human Development, Biostatistics and Bioinformatics Branch, Rockville, Maryland, USA; 4 Division of Laboratory Sciences, National Center for Environmental Health, Centers for Disease Control and Prevention, Atlanta, Georgia, USA; 5 Department of Social and Preventive Medicine, University at Buffalo, Buffalo, New York, USA

**Keywords:** anovulation, cadmium, lead, menstrual cycle, mercury, reproductive hormones

## Abstract

Background: Metals can interfere with hormonal functioning by binding at the receptor site and through indirect mechanisms; thus, they may be associated with hormonal changes in premenopausal women.

Objectives: We examined the associations between cadmium, lead, and mercury, and anovulation and patterns of reproductive hormones [estradiol, progesterone, follicle-stimulating hormone (FSH), luteinizing hormone] among 252 premenopausal women 18–44 years of age who were enrolled in the BioCycle Study in Buffalo, New York.

Methods: Women were followed for up to two menstrual cycles, with serum samples collected up to eight times per cycle. Metal concentrations were determined at baseline in whole blood by inductively coupled mass spectroscopy. Marginal structural models with stabilized inverse probability weights and nonlinear mixed models with harmonic terms were used to estimate the effects of cadmium, lead, and mercury on reproductive hormone levels during the menstrual cycle and anovulation.

Results: Geometric mean (interquartile range) cadmium, lead, and mercury levels were 0.29 (0.19–0.43) μg/L, 0.93 (0.68–1.20) μg/dL, and 1.03 (0.58–2.10) μg/L, respectively. We observed decreases in mean FSH with increasing cadmium [second vs. first tertile: –10.0%; 95% confidence interval (CI), –17.3% to –2.5%; third vs. first tertile: –8.3%; 95% CI, –16.0% to 0.1%] and increases in mean progesterone with increasing lead level (second vs. first tertile: 7.5%; 95% CI, 0.1–15.4%; third vs. first tertile: 6.8%; 95% CI, –0.8% to 14.9%). Metals were not significantly associated with anovulation.

Conclusions: Our findings support the hypothesis that environmentally relevant levels of metals are associated with modest changes in reproductive hormone levels in healthy, premenopausal women.

Exogenous factors may affect hormonal function, and several epidemiologic studies in peri- and postmenopausal women have suggested that metals alter hormone levels (Gallagher et al. 2010; Krieg 2007; Mendola et al. 2008; Nagata et al. 2005). However, evidence among premenopausal women is sparse (Gerhard et al. 1998; Jackson et al. 2008).

Environmental estrogens act through genomic pathways by binding to the estrogen receptor and initiating transcription of estrogen-activated genes, and through nongenomic pathways that involve signaling initiated in the cellular membrane (Tilghman et al. 2008). Divalent metals, including lead and mercury, can initiate the estrogen receptor by interacting with amino acids at the receptor binding site (Zhang et al. 2008), and both metals exert estrogenic changes in experimental models (Choe et al. 2003). Stoica et al. (2000) reported that cadmium may obstruct the hormone-binding domain of the estrogen receptor-α, thereby affecting subsequent transcriptional processes (Guével et al. 2000), but Silva et al. (2006) reported that cadmium did not have estrogenic properties based on other assays. Cross-sectional data are inadequate to assess hormonal patterns during the menstrual cycle (Howards et al. 2009), especially in association with environmental exposures that may alter hormone levels at certain times of the menstrual cycle but not others. Traditional approaches fail to capture the multiple physiologic parameters during a regular menstrual cycle and do not account for the complex interplay among reproductive hormones, for which traditional methods to control for confounding are inadequate. Further, evaluating hormonal differences from the peak to nadir and shifts in hormonal peaks can provide valuable insight into subclinical changes in hormonal function. Hormonal alterations may increase anovulation, with effects on fertility (Aksel et al. 1976; Lutoslawska et al. 2006), but anovulation has not been assessed among women who have been exposed to metals.

Our objective in the present study was to estimate associations between biomarkers of metal exposures (cadmium, lead, and mercury) and *a*) patterns of reproductive hormones [estradiol, progesterone, luteinizing hormone (LH), and follicle-stimulating hormone (FSH)], including mean change, amplitude, and phase shift across the cycle; *b*) mean change in reproductive hormones over the menstrual cycle while accounting for dependence between hormones; and *c*) anovulation among healthy, premenopausal women.

## Materials and Methods

*Study participants.* The BioCycle Study enrolled 259 healthy, regularly menstruating women (18–44 years of age) for up to two cycles to determine associations among biomarkers of oxidative stress, antioxidants, and hormonal levels during the menstrual cycle (Wactawski-Wende et al. 2009). Women who self-reported a menstrual cycle length between 21 and 35 days, who were not trying to conceive, and who had not used hormonal contraception in the past 3 months were included in the study. Women were followed prospectively for one (*n* = 9) or two (*n* = 250) menstrual cycles. The present analysis includes 252 women. Metals were measured from a single whole-blood sample that was collected at enrollment, and hormones were measured in blood samples collected up to eight times per cycle, with 94% of women completing seven or eight clinic visits per cycle. Data collection occurred from 2005 to 2007 at the University at Buffalo in New York. The University at Buffalo Health Sciences Institutional Review Board (IRB) approved the study, and all participants provided written informed consent. Under a reliance agreement, the National Institutes of Health depends on the designated IRB of the University at Buffalo for review, approval, and continuing oversight of its human subject research for the BioCycle Study.

*Exposure assessment.* At the enrollment visit, which occurred an average of 16 days before the first clinic visit, whole blood was collected in purple-top Vacutainer tubes (Becton, Dickinson and Company, Franklin Lakes, NJ) that contained EDTA and that were prescreened for trace metals. After the samples were collected, they were refrigerated and later sent to the CDC’s Division of Laboratory Sciences, National Center for Environmental Health, for lead, cadmium, and mercury assessment by inductively coupled plasma mass spectrometry. Mercury levels represent the total concentration of mercury in blood from all relevant forms of mercury (e.g., inorganic, methyl). The limits of detection (LODs) for cadmium, lead, and mercury were 0.20 μg/dL (25% < LOD), 0.25 μg/dL (0% < LOD), and 0.30 μg/dL (12% < LOD). Lab-reported values < LOD were used without substitution to minimize potential bias (Craig et al. 2003; Richardson and Ciampi 2003; Schisterman et al. 2006).

*Biospecimen collection.* Fasting blood samples for hormone measurements were collected in the morning to minimize diurnal variation. Participants used Clearblue Easy fertility monitors (Inverness Medical, Waltham, MA) to assist in scheduling midcycle visits (Howards et al. 2009), beginning on the sixth day after the start of menses and continuing daily testing until either the monitor indicated an LH surge or 20 test days had passed. If the monitor indicated peak fertility, the woman was instructed to come into the clinic that day and the next 2 days. Other visits were scheduled based on an algorithm that considered women’s reported cycle length. Visits were timed to correspond to early menstruation, mid- and late follicular phase, twice around expected ovulation, and early and late luteal phase.

*Outcomes assessment.* Reproductive hormones were measured in fasting serum samples collected in red-top tubes with no anticoagulant, spun, serum aliquoted, stored at –80°C, and shipped in batches that included a woman’s complete cycle samples. Estradiol was measured with radioimmunoassay. Progesterone, LH, and FSH were measured using solid-phase competitive chemiluminescent enzymatic immunoassays by Specialty Laboratory (Valencia, CA) on a DPC Immulite 2000 analyzer (Siemens Medical Solutions Diagnostics, Deerfield, IL). The interassay coefficients of variation reported by the laboratory for estradiol, LH, FSH, and progesterone were 9.7%, 4.8%, 4.8%, and 14.1%, respectively.

Cycles were classified as anovulatory if the progesterone level was ≤ 5 ng/mL throughout the entire cycle and if no serum LH peak was observed on the mid- or late-luteal-phase visit (Gaskins et al. 2009). Based on this definition, 42 of the 509 (8%) cycles were considered anovulatory, including 24 (57%) that occurred during the first cycle and 18 (43%) during the second. We also conducted a sensitivity analysis using a less restrictive definition of anovulation based on progesterone across the cycle of < 5 ng/g (Abdulla et al. 1983; Malcolm and Cumming 2003) that resulted in 65 cycles (13%) being classified as anovulatory.

*Covariate assessment.* At enrollment, women provided written consent and then were asked to provide a health and reproductive history and lifestyle information (e.g., smoking, alcohol intake), and anthropometric measurements were taken by trained staff. In addition, usual physical activity was assessed using the International Physical Activity Questionnaire (IPAQ) (Yang et al. 2004) and categorized according to standard IPAQ cut-points, and a food frequency questionnaire was used to estimate usual daily total energy, iron, shellfish, fish, and vegetable intakes during the previous 6 months (Fred Hutchinson Cancer Center, Seattle, WA).

*Statistical methods.* Descriptive statistics for continuous and categorical covariates were compared by tertiles of metal exposure and ovulatory status using analysis of variance, chi-square test, or Fisher’s exact test, as appropriate. Hormone levels were natural log transformed for normality. Metals were assessed as tertiles and continuous variables. Models were run separately for each metal. Potential confounders were identified (based on a review of the literature) and included age, body mass index (BMI), and race (white, black, Asian, other). We also evaluated smoking; income; education; physical activity; parity; dietary iron, fish, shellfish, and vegetables; and total calories as potential confounders; these factors were not included in the final models because they did not alter estimated associations by > 10% between metals and hormones or anovulation. Because of the small number of smokers, we restricted the sensitivity analyses to nonsmokers. One individual was excluded from continuous analyses of cadmium because her level was > 3 SDs from the mean.

We used generalized linear mixed models to estimate the association between metal exposures (modeled as continuous variables) and anovulation while accounting for dependence within women and cycles using SAS (version 9.2; SAS Institute Inc., Cary, NC). With these models, inferences are subject specific; the odds ratios correspond to a change in odds of anovulation associated with a one-unit change in blood metal level for an individual subject (Zeger et al. 1988).

We used nonlinear mixed effects models with harmonic terms (version 2.10.1; R Foundation for Statistical Computing, Vienna, Austria) to evaluate associations between metals (grouped according to tertiles) and patterns of reproductive hormones over one to two menstrual cycles, accounting for cyclical hormonal changes and complex between-subject variation (Albert and Hunsberger 2005). Outcomes for each hormone (natural-log–transformed estradiol, FSH, LH, and progesterone) included mean concentration, amplitude (the difference between nadir and peak hormone levels), and timing of shifts in the hormonal profile. Random effects for models of hormone means and amplitudes included a subject-specific term and, for each subject, a cycle-specific term. The number of harmonic terms used in each model was selected to minimize the Akaike information criteria (Akaike 1973) and to reflect the expected shape of the hormonal profile. Time was scaled such that time was set to 0 on the first day of an individual’s cycle, 0.5 on the day of ovulation, and 1.0 on the last day of the cycle, regardless of the actual calendar days. Models to estimate associations with tertiles of metal exposure were run separately for each metal and were adjusted for age (continuous), BMI (continuous), and race (white, black, Asian, other).

We used generalized linear mixed models with stabilized weights to estimate effects of metals (continuous) on natural-log–transformed hormone levels while appropriately accounting for confounding by hormone levels at other times during the cycle (Cole and Hernan 2008; Robins et al. 2000). Inverse probability of treatment weights were conditioned upon estradiol, progesterone, LH, FSH, race (white, black, Asian, other), age, and BMI. Adujsting for smoking (current vs. nonsmoker) and average calorie intake (continuous) did not appreciably alter the estimates; thus, these factors were not included in the final models. An α-level ≤ 0.05 denoted statistical significance. We assessed interactions between each pair of metals separately with all three metals included in one model using a *p* < 0.10 criterion.

## Results

*Metal exposure.* In Table 1, we summarize the BioCycle cohort population characteristics. Mean age of participants was 27 years, and mean BMI was 24 kg/m^2^. Most participants were white, unmarried, and nulliparous. Cadmium and lead were positively associated with age, whereas mercury was not. BMI was not associated with metal exposures. Higher blood cadmium and lead levels were more common among nonwhites, whereas blood mercury was associated with race—those who identified themselves as Asian had the highest mercury levels. Smoking was positively marginally associated with cadmium but not with lead or mercury. Women with anovulatory cycles were more likely to be nulliparous and unmarried. Twenty-three participants completed the study in nonconsecutive cycles, and their demographics were similar to those completing the study in consecutive cycles. Because metals and hormones were right-skewed, we calculated geometric means. Cadmium was positively correlated with lead (ρ = 0.12, *p* = 0.04) and mercury (ρ = 0.12, *p* = 0.05). Lead and mercury were not statistically correlated (ρ = 0.08, *p* = 0.19).

**Table 1:** BioCycle Study population characteristics, Buffalo, New York (2005–2007).

Characteristic	BioCycle Study population
Age (mean ± SD)		27.3 ± 8.2
BMI [kg/m^2^ (mean ± SD)]		24.1 ± 3.9
Calories [kcal/day (mean ± SD)]		1,608 ± 353
Follicular estradiol [pg/mL; GM (IQR)]*a*		33.2 (25.0–43.0)
Midcycle FSH [mIU/mL; GM (IQR)]*b*		6.8 (4.7–10.2)
Midcycle LH [ng/mL; GM (IQR)]*b*		11.6 (5.6–15.0)
Luteal progesterone [ng/mL; GM (IQR)]*c*		6.2 (4.8–12.2)
Cadmium [μg/L; GM (IQR)]*d*		0.29 (0.19–0.43)
Lead [μg/dL; GM (IQR)]*d*		0.93 (0.68–1.20)
Mercury [μg/L; GM (IQR)]*d*		1.03 (0.58–2.10)
Anovulatory cycles (*n*)		
0		221
1		25
2		6
Race (*n*)		
White		150
Black		51
Asian		36
Other		15
Smoking (*n*)		
No/former		242
Current		10
Marital status (*n*)		
Ever married		80
Physical activity		
Low		24
Medium		89
High		139
Parity (*n*)		
Nulliparous		182
Education (*n*)		
> High school		221
IQR, interquartile range. **a**Geometric mean estradiol levels (IQR) measured during early follicular phase, cycle day 2. **b**Geometric mean FSH and LH levels (IQR) measured during midcycle, cycle day 13. **c**Geometric mean progesterone levels (IQR) measured during luteal phase, cycle day 22. **d**Geometric mean (IQR) blood metal levels.		

**Figure 1:**
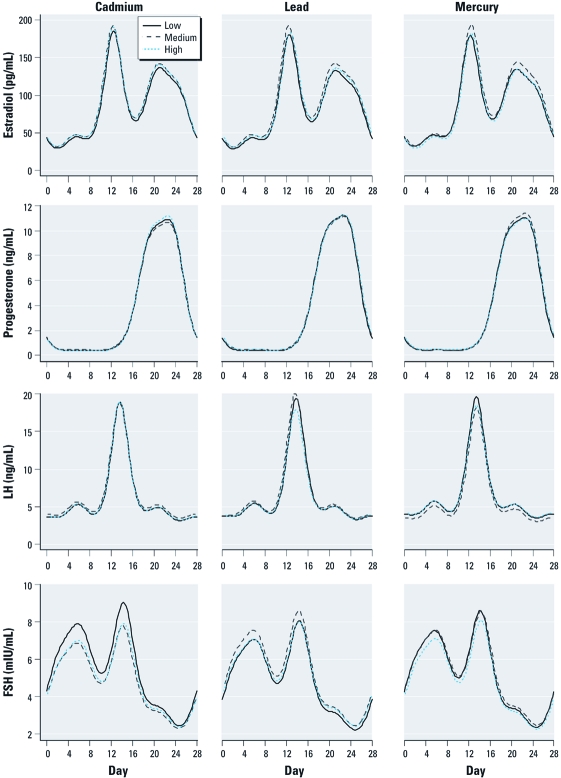
Metal exposure and reproductive hormone changes in a standardized menstrual cycle with four-term nonlinear harmonic models, BioCycle Study (*n* = 234). Cadmium tertiles: low, 0.04–0.23 μg/L; medium, 0.24–0.36 μg/L; high, 0.37–3.10 μg/L. Lead tertiles: low, 0.30–0.71 μg/dL; medium, 0.72–1.10 μg/dL; high, 1.11–6.20 μg/dL. Mercury tertiles: low, 0.00–0.74 μg/L; medium, 0.75–1.50 μg/L; high, 1.51–9.90 μg/L.

*Pituitary reproductive hormones.* Using nonlinear mixed models with four harmonic terms, cadmium exposure was associated with –10% [95% confidence interval (CI), –17.3% to –2.5%] and –8.3% (95% CI, –16.0% to 0.1%) decreased mean FSH levels for the highest and middle tertiles, respectively, compared with the lowest exposed tertiles (Table 2). Cadmium was associated with changes in FSH amplitude (peak to nadir difference) of –0.02 (95% CI, –0.05 to 0.01) and –0.02 (–0.05 to 0.01) for the highest and middle tertiles, respectively, compared with the lowest. Lead was associated with increased FSH means of 8.0% (95% CI, –0.9% to 17.7%) for the middle tertile and 3.6% (95% CI, –5.3% to 13.3%) for the upper tertile, compared with the lowest. Mercury was associated with a change in LH for the middle tertile of –11.7% (95% CI, –20.0% to –2.5%) compared with the lowest. Figure 1 displays the hormonal patterns for estradiol, progesterone, LH, and FSH by tertile of metal exposure adjusted for race, age, and BMI (plots show tertiles for white women of mean age and BMI) and shows a decreased mean and amplitude for FSH in relation to cadmium exposure and decreased LH for the middle tertile of mercury.

**Table 2:** Nonlinear mixed models with four harmonic terms for metals (low = referent) and natural-log–transformed hormones among women with ovulatory cycles (*n* = 234), BioCycle Study (2005–2007).*a*

Mean % (95% CI)			Amplitude (95% CI)			Phase shift [day*b* (95% CI)]		
Characteristic	Medium	High	Medium	High	Medium	High
Estradiol (pg/mL)												
Cadmium (μg/L)		5.5 (–3.5 to 15.5)		5.1 (–4.1 to 15.2)		–0.01 (–0.06 to 0.04)		–0.02 (–0.06 to 0.05)		–0.12 (–0.29 to 0.02)		–0.04 (–0.20 to 0.12)
Lead (μg/dL)		8.2 (–1.2 to 18.6)		4.7 (–4.7 to 15.2)		–0.01 (–0.06 to 0.04)		–0.02 (–0.7 to 0.03)		–0.09 (–0.24 to 0.05)		0.14 (–0.01 to 0.29)
Mercury (μg/L)		5.5 (–3.5 to 15.5)		–2.4 (–10.9 to 6.9)		0.02 (–0.02 to 0.07)		0.03 (–0.02 to 0.8)		0.15 (0.01 to 0.29)		0.18 (0.03 to 0.33)
FSH (mIU/mL)												
Cadmium (μg/L)		–10.0 (–17.3 to –2.5)		–8.3 (–16.0 to 0.1)		–0.02 (–0.05 to 0.01)		–0.02 (–0.05 to 0.01)		–0.13 (–0.31 to 0.05)		0.09 (–0.09 to 0.28)
Lead (μg/dL)		8.0 (–0.9 to 17.7)		3.6 (–5.3 to 13.3)		–0.01 (–0.03 to 0.02)		–0.02 (–0.04 to 0.01)		–0.06 (–0.25 to 0.12)		–0.02 (–0.21 to 0.18)
Mercury (μg/L)		1.7 (–6.6 to 10.6)		–5.1 (–12.9 to 3.4)		–0.01 (–0.3 to 0.02)		–0.01 (–0.03 to 0.07)		0.15 (–0.03 to 0.33)		0.14 (–0.05 to 0.32)
LH (ng/mL)												
Cadmium (μg/L)		7.0 (–3.1 to 18.4)		1.0 (–9.0 to 12.1)		–0.02 (–0.05 to 0.01)		0.00 (–0.03 to 0.03)		–0.07 (–0.27 to 0.13)		–0.03 (–0.23 to 0.17)
Lead (μg/dL)		5.1 (–5.1 to 16.4)		–0.5 (–10.5 to 10.7)		–0.01 (–0.03 to 0.02)		–0.02 (–0.04 to 0.01)		–0.16 (–0.36 to 0.03)		–0.11 (–0.32 to 0.10)
Mercury (μg/L)		–11.7 (–20.0 to –2.5)		0.4 (–9.2 to 11.1)		0.02 (–0.01 to 0.04)		–0.02 (–0.05 to 0.01)		0.02 (–0.19 to 0.21)		0.01 (–0.19 to 0.22)
Progesterone (ng/mL)												
Cadmium (μg/L)		2.7 (–4.3 to 10.2)		–2.3 (–9.2 to 5.1)		–0.04 (–0.11 to 0.03)		0.03 (–0.04 to 0.11)		–0.07 (–0.18 to 0.03)		–0.03 (–0.13 to 0.07)
Lead (μg/dL)		7.5 (0.1 to 15.4)		6.8 (–0.8 to 14.9)		0.07 (0.01 to 0.15)		–0.06 (–0.13 to 0.01)		0.04 (–0.06 to 0.15)		0.15 (0.05 to 0.26)
Mercury (μg/L)		1.4 (–5.4 to 8.8)		3.6 (–3.6 to 11.3)		0.01 (–0.06 to 0.09)		–0.04 (–0.11 to 0.03)		0.09 (–0.00 to 0.19)		0.13 (0.03 to 0.24)
**a**Time scale for mean and amplitude results ranged from 0 to 1; time scale for phase shift is a 28-day cycle. Data are adjusted for age (continuous), race (white, black, Asian, other), and BMI (continuous). Cadmium tertiles: low, 0.03–0.23 μg/L; medium, 0.24–0.36 μg/L; high, 0.37–3.10 μg/L. Lead tertiles: low, 0.30–0.72 μg/dL; medium, 0.73–1.10 μg/dL; high, 1.11–6.20 μg/dL. Mercury tertiles: low, 0.00–0.74 μg/L; medium, 0.75–1.50 μg/L; high, 1.51–9.00 μg/L. **b**Proportion of 24 hr day.												

*Ovarian reproductive hormones.* Using nonlinear mixed models with four harmonic terms, we observed that metals were not statistically significantly associated with estradiol levels. However, cadmium and lead levels were consistently but not statistically significantly associated with increased mean and decreased amplitude of estradiol levels (Table 2). The amplitude for estradiol was decreased, although not statistically significant, for increased cadmium and lead exposure (Table 2). Mercury was not consistently associated with changes in direction of estradiol mean, although increasing exposure was associated with delayed rises of estradiol of 0.15 (95% CI, 0.01 to –0.29) and 0.18 (95% CI, 0.03 to –0.33) equivalent to approximately 3–4 hr. Cadmium and lead were not associated with phase shifts in estradiol. Mean progesterone was 7.5% (95% CI, 0.1–15.4%) higher for participants in the middle versus low tertile of lead and 6.8% (95% CI, –0.8% to 14.9%) higher for the high versus low tertile (Table 2). Lead was associated with a delayed rise of progesterone, with a 0.15-day shift compared with the lowest tertile (95% CI, 0.05–0.26). Mercury was similarly associated with a later progesterone rise, with shifts of 0.09 (95% CI, < –0.01 to 0.19) and 0.13 (95% CI, 0.03–0.24) days for the middle and high tertiles compared with the lowest. Cadmium was not consistently associated with changes in progesterone mean or amplitude.

*Marginal structural models.* Weighted linear mixed models that were adjusted for changing hormone levels across the cycle indicated that cadmium, lead, and mercury were not statistically significantly associated with mean log-transformed estradiol, progesterone, LH, or FSH concentrations (Table 3).

**Table 3:** Weighted linear mixed models of blood cadmium, lead, and mercury (continuous) on reproductive hormones (natural log transformed) in the BioCycle Study (2005–2007).

Log-transformed hormone	Cadmium		Lead		Mercury	
	*n*	β-Coefficient	95% CI	β-Coefficient	95% CI	β-Coefficient	95% CI
Estradiol (pg/mL)		218	0.16	–0.02 to 0.34		0.03	–0.05 to 0.11		0.001	–0.03 to 0.04
FSH (mIU/mL)		218	0.004	–0.14 to 0.15		–0.01	–0.07 to 0.06		0.01	–0.02 to 0.03
LH (ng/mL)		218	0.11	–0.07 to 0.29		0.02	–0.06 to 0.10		0.01	–0.02 to 0.05
Progesterone (ng/mL)		218	–0.01	–0.26 to 0.23		0.06	–0.04 to 0.17		–0.04	–0.08 to 0.01
Data adjusted for BMI (continuous), race (white, black, Asian, other), and age (continuous) and weighted using inverse probability of treatment weights.										

*Anovulation.* Metals were not statistically significantly associated with anovulation. Odds ratios for anovulation per 1 μg/L cadmium, 1 μg/dL lead, and 1 μg/L mercury were 1.29 (95% CI, 0.20–8.47), 1.20 (95% CI, 0.62–2.34), and 1.12 (95% CI, 0.85–1.48), respectively, adjusted for age, race, and BMI. After restricting the analyses to persons who had never smoked > 100 cigarettes (*n* = 195), the odds ratios for cadmium, lead, and mercury were 1.66 (95% CI, 0.22–12.30), 0.93 (95% CI, 0.39–2.91), and 1.07 (95% CI, 0.79–1.46), respectively. Additional sensitivity analyses conducted using a less conservative definition of ovulation (progesterone ≤ 5 ng/mL, n = 65 cycles) yielded consistent results, and the odds ratios for cadmium, lead, and mercury were 1.39 (95% CI, 0.63–3.07), 1.50 (95% CI, 0.62–3.60), and 1.29 (95% CI, 0.48–3.47).

## Discussion

Our findings provide some evidence that low levels of metals may be associated with modest hormonal variation in healthy, premenopausal women. At environmentally relevant levels, we observed a positive association between mean progesterone and lead levels above the lowest tertile and declines in FSH mean concentration and amplitude with cadmium levels above the lowest tertile. The combination of increasing mean and decreasing amplitude we observed for lead and FSH and evidence of increased mean estradiol with cadmium and lead may indicate the start of resistance to ovulation, similar to what naturally occurs among perimenopausal women, where greater estradiol and FSH levels are needed to achieve ovulation (Santoro et al. 1996). However, we observed no significant associations between metals and anovulation. Our findings highlight the need for additional research on ovulatory and hormonal function in premenopausal women.

We observed slight differences between the marginal structural models and nonlinear mixed models with harmonic terms. Nonlinear models enable detection of changes in amplitude and phase shift, in addition to mean differences. Marginal structural models assess only mean differences and are less efficient because of the weight term. The discrepancies could possibly be explained by differences in efficiency and the parameters assessed between the two approaches. Alternatively, time-dependent confounding by endogenous hormone levels could explain the disparity, because methods to weight harmonic models have not been developed.

We built upon previous studies that did not take timing of hormonal measurements into account but suggested a link between metal levels and hormones. Our findings differed from a study reporting a positive association between cadmium and FSH in perimenopausal women (Gallagher et al. 2010). However, our study population was premenopausal, and hormonal measurements were timed over the course of the menstrual cycle, whereas hormone measurements in Gallagher et al. (2010) were not timed to the menstrual cycle, and cadmium was measured in urine reflecting a longer exposure time period than blood. Cadmium levels were lower among BioCycle participants (geometric mean, 0.29 μg/L) than among similar populations (geometric mean, 0.42 μg/L) (Jackson et al. 2008), possibly because of the low prevalence of smoking (Järup and Akesson 2009; Satarug and Moore 2004). Further, we conducted sensitivity analyses restricting to never smokers and adjusted all other analyses for smoking, and the results were similar (data not shown). We found that lead was positively, although not statistically significantly, associated with estradiol, similar to previous findings in a case–control study of infertility (Chang et al. 2006). Our findings that lead was positively associated with progesterone and positively but not statistically significantly associated with estradiol are consistent with a recent study of lead and inhibin B, a marker of follicular development. In this study, Gollenberg et al. (2010) found that high levels of lead were associated with decreased odds of high inhibin B, which indicates a possible pubertal delay. The observed association with elevated ovarian hormones and lead could possibly indicate that greater levels of ovarian hormones are necessary for normal reproductive hormonal function. In one study of placental metals and hormones, Stasenko et al. (2010) found no association between lead and progesterone. Our findings that suggest a positive association between progesterone and lead differ from those of studies of higher levels of lead exposure in monkeys that showed inverse associations with progesterone (Foster et al. 1996; Laughlin et al. 1987). A cross-sectional study of women with repeated miscarriage reported that follicular-phase estradiol was positively associated with mercury and lead (Gerhard et al. 1998), and our findings were similar for lead, although not statistically significant. Our study suggests that environmental exposures to metals may be associated with modest hormonal effects in healthy, regularly menstruating women, a population not often assessed in relation to environmental or hormonal outcomes.

Experimental evidence supports our findings of a positive association between lead and mean progesterone and an inverse association between cadmium and mean FSH. Evidence suggests that lead affects the hypothalamic–pituitary–gonadal axis by diminishing expression of the steroidogenic acute regulatory protein gene (Srivastava et al. 2004). Further, lead exposure to ovarian granulosa cells induces peptides related to proliferation (cyclin B1) and apoptosis (caspace-3) (Kolesarova et al. 2010). P450 cholesterol side chain cleavage plays a role in progesterone synthesis, and lead could influence progesterone via this mechanism (Zhang and Jia 2007). Our findings that cadmium was associated with decreased FSH mean and amplitude are consistent with findings that cadmium may inhibit P450 cholesterol side chain cleavage (Kawai et al. 2002), thereby disrupting ovarian hormone synthesis or, alternatively, findings that cadmium may interact directly with the estrogen receptor (Garcia-Morales et al. 1994).

Women who participated in BioCycle were not occupationally exposed to metals or otherwise highly exposed. Cadmium exposure is primarily due to cigarette smoke, and for nonsmoking individuals, exposure is mainly from diet, particularly from consuming shellfish, organ meats, leafy vegetables, and grains grown in soil contaminated with cadmium (Groten and van Bladeren 1994). Exposure tends to vary by race/ethnicity (Mijal and Holzman 2010). Cadmium is stored in the kidney, and when measured in urine its half-life is estimated between 15 and 30 years, compared with several months in blood; thus, differences between our study and others may be attributable to quantifying exposure reflecting different time periods [Agency for Toxic Substances and Disease Registry (ATSDR) 1999b]. Although lead exposure in the United States has declined in recent years, it remains widespread and comes from inhalation and ingestion (Pirkle et al. 1994; Mielke and Reagan 1998). Lead is stored for years to decades in bone, and in blood it has an estimated half-life of 1–2 months. Blood lead can be thought of as a steady-state marker of lead exposure (Rabinowitz 1991; Hu et al. 2007). The preponderance of lead sequestered in bone, or the lower levels of lead exposure among the study participants compared with previous studies that focused on older and more highly exposed populations, may explain our modest findings in relation to lead exposure. Although inorganic mercury is stored in the kidney, methylmercury passes through the blood–brain barrier, is stored in body tissues, and has an estimated half-life of 2–4 months (Aberg et al. 1969); fish consumption is the primary exposure source (ATSDR 1999a). Although measuring metals in blood reflects exposure on the order of months, these exposures are likely to reflect a steady state of diet and ambient exposures. Our modest findings may be attributable to the limited range of exposure among BioCycle participants. Lead and mercury levels in our study were lower than levels reported in premenopausal women in the National Health and Nutrition Examination Survey (NHANES 1999–2002) in New York (geometric mean: 0.87 vs. 1.79 μg/dL for lead, and 1.10 vs. 2.73 μg/L for mercury) (Jackson et al. 2008). These differences could be due in part to lower smoking prevalence among BioCycle participants [4% vs. national average 25% (Mijal and Holzman 2010)]. Geometric mean cadmium levels in BioCycle participants, however, were similar to those among never-smoking premenopausal women in NHANES (0.30 vs. 0.29 μg/L) (Mijal and Holzman 2010). Alternatively, rigorous selection for women with normal reproductive function, although intended to minimize confounding, may have omitted women with greater levels of metals, if such associations were present, indicating that stronger associations may be seen in other populations. Further, to determine if metal exposure was associated with completing follow-up in consecutive cycles, we examined whether the distribution of metals differed among the 23 women with nonconsecutive cycles and found no differences in the results.

Our work was subject to several limitations. Assessing metals in blood generally provides short-term exposure history. Cadmium measured in urine is widely accepted as a marker of long-term exposure (ATSDR 1999b). However, because we believe that women who participated in the BioCycle Study had environmental exposures to metals that likely came from inhalation and diet, their short-term biomarker levels may represent a steady state of exposure. Further, detecting modest hormonal effects is difficult in premenopausal women because of endogenous hormonal variation, a challenge inherent to studying women of reproductive age. However, the use of fertility monitors improved our ability to capture phases of the menstrual cycle with maximal hormonal variability. We cannot rule out possible unmeasured confounding factors, particularly for status of cadmium and iron (Vahter et al. 2002), an essential metal that we did not measure. Iron could be considered a confounder because cadmium update in the gastrointestinal tract is inversely related to iron levels, and iron levels are associated with hormone levels. Although our use of weighted models appropriately accounted for possible time-varying confounding factors affected by prior exposure, these models are less precise, which may explain why those results were not statistically significant. Further, our results may not be broadly generalizable, because the BioCycle Study represented a highly selected population.

To the best of our knowledge, this is the first study to assess anovulation and reproductive hormone levels over time in relation to metal exposure in healthy, premenopausal women. We measured metals and hormones using the most accurate and precise methods available, and we measured reproductive hormones at multiple well-timed visits across two menstrual cycles. Further, we selected women for the BioCycle Study to include an ethnically diverse, healthy population, with no reported history of a range of reproductive health conditions, minimizing the impact of factors known to be associated with reproductive function. Information was available on multiple potential confounding factors. In addition, we used methods to evaluate specific components of the hormonal profile and compared those results with weighted linear mixed models that account for time-varying confounding affected by prior exposures. These analytical tools represent an improvement over previous studies in evaluation of hormonal patterns across the menstrual cycle and provide information on various parameters of the hormonal profile. Future work to develop nonlinear mixed effects models that account for time-varying confounding factors will improve our understanding of the interplay between reproductive hormones and chemical exposures in premenopausal women.

## Conclusions

We demonstrated that lead levels above the lowest tertile were associated with higher mean progesterone levels, whereas cadmium levels above the lowest tertile was associated with decreased FSH mean and amplitude. We observed these associations among healthy, regularly menstruating women at environmentally relevant exposure levels. Because hormone levels in women are considered risk factors for cardiovascular disease (Rossouw et al. 2002) and breast and ovarian cancer (Brinton et al. 1988), understanding how endogenous levels of metals contribute to variation in reproductive hormone levels provides additional insight and opportunities for prevention. To our knowledge, this is the first study to examine anovulation in relation to metal exposures. Additional research on the effects of metals, including long-term biomarkers of exposure and longitudinal evaluation of ovulation and hormonal function, in premenopausal women is warranted.
